# FLAIR hyperintense cortical lesions in myelin oligodendrocyte glycoprotein-associated encephalitis with seizures in children: a retrospective single-center case series

**DOI:** 10.3389/fimmu.2025.1563481

**Published:** 2025-07-16

**Authors:** Kang Liu, Weixiu Wang, Mei Jin, Fang Chen, Wei Wang, Xiaohan Liu, Suzhen Sun

**Affiliations:** ^1^ First Department of Neurology, Hebei Children’s Hospital, Shijiazhuang, China; ^2^ The Key Laboratory of Pediatric Epilepsy and Neurological Disorders of Hebei Province, Hebei Children's Hospital, Shijiazhuang, China; ^3^ Department of Radiology, Hebei Children’s Hospital, Shijiazhuang, China

**Keywords:** cortical encephalitis, seizures, myelin oligodendrocyte glycoprotein antibody, brain magnetic resonance imaging, children

## Abstract

**Background and purpose:**

In recent years, the number of case reports concerning fluid-attenuated inversion recovery (FLAIR) hyperintense cortical lesions in myelin oligodendrocyte glycoprotein (MOG) -associated encephalitis with seizures (FLAMES) has been gradually increasing. However, within the pediatric demographic, there remains a lack of related serial reports. This study was designed to characterize the clinical features and prognosis of FLAMES in pediatric patients.

**Methods:**

We reviewed the medical records of children diagnosed with FLAMES from January 2019 to July 2024 and retrospectively analyzed their clinical manifestations, brain magnetic resonance imaging (MRI) findings, laboratory results, treatments, and outcomes.

**Results:**

Among the 123 children diagnosed with MOG antibody-associated diseases (MOGAD), 9 (7.3%) met the inclusion criteria for FLAMES. The median onset age was 9 years (range: 8-14), and the male-to-female ratio was 5:4. The most common clinical symptoms included seizures (9/9, with 6 experiencing focal seizures), altered mental status (6/9), headache (5/9), fever (4/9), and focal neurological deficits (3/9). Furthermore, three patients presented with status epilepticus, two with cranial nerve involvement (central facial paralysis and lingual paralysis), and two with Todd’s palsy. Seven patients had cerebrospinal fluid (CSF) pleocytosis (median count: 58/µL, range: 12-143/µL); two had elevated CSF pressure (range: 240–280 mmH2O); and one had mildly elevated CSF protein levels (0.46 g/L). All patients had normal CSF glucose levels. Abnormal electroencephalogram (EEG) findings were detected in seven patients: epileptic seizures (3/7), interictal discharges (6/7), and slow background activity (3/7). Unilateral cortical hyperintense lesions were observed in all nine cases on FLAIR imaging of brain MRI, affecting the frontal (8/9), parietal (3/9), temporal (2/9), and occipital (1/9) lobes. Five patients exhibited distinct linear enhancement of the corresponding cerebral sulci and/or meninges on gadolinium-enhanced brain MRI. All patients received immunotherapy, and six were administered anti-seizure medicines (ASMs). Each child achieved a satisfactory outcome and remained relapse-free at the final follow-up.

**Conclusion:**

FLAMES exhibit an age-dependent pattern. Epileptic seizures are the most common clinical symptom, with focal seizures being the predominant type. FLAIR-hyperintense cortical lesions typically present unilaterally, predominantly affecting the frontal lobes. Enhancement of the corresponding cerebral sulci and/or meninges may be a distinctive feature in children. For children with epilepsy, in the presence of recurrent seizures without identifiable triggers, especially when cortical lesions are detected in cranial MRI, consideration should be given to the possibility of FLAMES.

## Introduction

Myelin oligodendrocyte glycoprotein(MOG), a protein located on the surface of oligodendrocytes, plays a crucial role in maintaining microtubule stability, ensuring myelin structural integrity through adhesion, and facilitating interactions between myelin and the immune system (1). In recent years, MOG-IgG has been increasingly detected in various clinical phenotypes. Given the significant heterogeneity in clinical characteristics and brain magnetic resonance imaging (MRI) manifestations, along with the immunopathological mechanism that is distinct from those of other central nervous system demyelinating diseases (2, 3), it has been gradually recognized as a new entity of immune-inflammatory diseases, now termed MOG antibody-associated diseases (MOGAD) (4).

“FLAMES” (FLAIR-hyperintense Cortical Lesions in MOG-associated Encephalitis with Seizures), a distinct clinical-imaging syndrome initially described by Ogawa in 2017 (5) and subsequently named by Burdham in 2019 (6), has been recognized as a rare phenotype of MOGAD. The hallmark features of this MOGAD entity are seizures accompanied by cortical hyperintensities, which are prominently visible on brain MRI T2-weighted fluid-attenuated inversion recovery (FLAIR) sequences and correlate with symptoms corresponding to cortical lesions. In addition to cortical signal abnormalities, MRI reveals cortical swelling and leptomeningeal enhancement. Given the predominantly unilateral cortical lesions, it can be easily mistaken for encephalitis, meningitis, central nervous system (CNS) vasculitis, and mitochondrial encephalopathy (especially MELAS: mitochondrial encephalopathy, lactic acidosis, and stroke-like episodes); Moreover, pediatric patients are more prone to multifocal phenotypes than adults (7), and the involvement of white matter and deep gray matter can significantly increase the risk of misdiagnosis.

To date, the quantity of clinical reports on FLAMES is rather limited, particularly those concerning the pediatric population. Consequently, the clinical characteristics and outcomes are not yet fully understood, and the factors influencing disease progression and long-term prognosis remain undetermined. In this study, we evaluate nine pediatric patients diagnosed with FLAMES at a single center in Northern China.

## Patients and methods

This study conducted a retrospective review of clinical data from children hospitalized at Hebei Children’s Hospital between January 2019 and July 2024. The inclusion criteria were as follows: (i) the first acute episode of MOGAD; (ii) met the diagnostic criteria of International MOGAD Expert Group in 2023; (iii) positive serum MOG-IgG, with negative aquaporin-4 (AQP4) -IgG and other immune antibodies (including NMDAR, AMPA1, AMPA2, GABAB, LGI1,CASPR2, GAD65, mGluR5, GFAP, and Hu, Yo, Ri, Ma2, CV2, Amphiphysin, ANNA-3, Tr, PCA-2, GAD antibodies); (iv) patients presenting with encephalitis accompanied by seizures and exhibiting solely cortical hyperintense lesions on the FLAIR sequence of MRI, without any other lesions; and (v) exclusion of cortical encephalitis due to other etiologies, such as intracranial infections, CNS vasculitis, and genetic metabolic diseases.

The clinical dataset included age, gender, hospitalization duration, presenting symptoms, laboratory parameters, brain MRI findings, treatment, and outcomes. The Expanded Disability Status Scale (EDSS) was utilized to assess disease severity. All cerebral magnetic resonance imaging scans were performed using a 3T field strength magnet. Serum MOG antibodies were detected using live cell-based assays, while cerebrospinal fluid (CSF) MOG antibodies were detected using fixed cell-based assays; based on laboratory assay parameters, we use a titer cutoff of 1:100 for high positive and 1:10 to 1:32 for low positive. After discharge, all patients were followed up through outpatient visits.

The study received approval from the Medical Ethics Committee of Hebei Children’s Hospital, which is affiliated with Hebei Medical University. Informed consent was obtained from parents or legal guardians of the participating children.

## Results

### Demographics and clinical features

In total, 123 children met the diagnostic criteria of MOGAD, among whom 9 cases (9/123, 7.32%) met the inclusion criteria and were diagnosed with FLAMES for the first time. The demographic and clinical characteristics of these nine patients are detailed in **Table 1**. The median onset age was 9 years (IQR 8.0-10.5), with a slight male predominance of 5:4. Only one child had a history of epilepsy but was seizure-free on oral levetiracetam, whereas the remaining eight had no prior history of central nervous system (CNS) diseases.

**Table 1:** Detailed demographic and clinical characteristics of 9 patients.

Variables	Patient 1	Patient 2	Patient 3	Patient 4	Patient 5
Demographics					
Age (y)	8	8	8	9	11
Sex	M	M	F	M	M
Clinical features					
Seizure type	FS	FS	FS	FBTCS	FS
Cluster seizures	✝	━	✝	✝	━
Status epilepticus	✝	━	━	✝	━
Altered mental status	✝	━	✝	✝	✝
Fever	✝	✝	━	━	✝
Headache	✝	━	━	━	✝
Cranial nerve paralysis	✝	✝	━	━	━
Focal neurologic deficits	━	✝	━	━	━
Laboratory features					
CSF pressure (mmH_2_O)	125	150	170	140	240
CSF WBC (cells/mm^3^)	64	42	5	32	12
CSF lymphocyte (%)	53.2	66.7	40	87.5	100
CSF protein (g/L)	0.23	0.35	0.18	0.26	0.23
CSF glucose (mmol/L)	3.83	3.47	3.14	3.14	3.44
Serum MOG-IgG	1:320	1:100	1:32	1:100	1:32
CSF MOG-IgG	1:10	━	━	1:10	━
CSF Oligoclonal bands	✝	━	━	✝	━
MRI features					
Cortical FLAIR hyperintensity	R Fr/P	L Fr	R Fr	R Fr	L Fr
DWI diffusion restriction	✝	✝	✝	✝	✝
Cerebral sulcus and/or Meningeal enhancement	ND	✝	✝	✝	✝
Other abnormality	━	━	✝	━	━
EEG features					
Slow background activity	━	✝	━	━	━
Interictal spike wave	━	✝	━	━	━
**Treatment latency (days)**	4	5	14	7	8
Treatment					
Immunotherapy (IVIG, IVMP+OP)	✝	✝	✝	✝	✝
Anti-infective drugs	✝	✝	━	✝	✝
ASM	PER	PER	/	LEV	LTG
**Length of stay (days)**	23	19	21	24	26
Outcome					
Follow-up duration (month)	5	6	18	32	36
Recovery	✝	✝	✝	✝	✝
Seizure free	✝	✝	✝	✝	✝
Last cranial MRI/time interval(month)	Improved/5	Improved/6	Improved/2	Normal/33	Improved/4
Last serum MOG-IgG/	1:100	1:32	━	1:320	━
time interval(month)	/8	/9	/3	/33	/6
EDSS score (worse/last follow-up)	3.5/1.0	4.0/1.0	2.0/0	3.0/0	2.0/0
Relapse	━	━	━	━	━
Variables	Patient 6	Patient 7	Patient 8	Patient 9	
Demographics					
Age (y)	9	9	10	14	
Sex	M	F	F	F	
Clinical features					
Seizure type	UTCS	TS	FS , TS	FS	
Cluster seizures	━	━	━	✝	
Status epilepticus	━	━	━	✝	
Altered mental status	━	✝	━	✝	
Fever	━	✝	━	━	
Headache	✝	━	✝	✝	
Cranial nerve paralysis	━	━	━	━	
Focal neurologic deficits	✝	━	✝	━	
Laboratory features					
CSF pressure (mmH_2_O)	280	160	135	110	
CSF WBC (cells/mm^3^)	58	143	79	1	
CSF lymphocyte (%)	86.2	88.1	84.8	0	
CSF protein (g/L)	0.26	0.27	0.46	0.25	
CSF glucose (mmol/L)	3.66	2.52	3.02	3.16	
Serum MOG-IgG	1:32	1:320	1:32	1:32	
CSF MOG-IgG	━	1:32	━	━	
CSF Oligoclonal bands	━	━	✝	━	
MRI features					
Cortical FLAIR hyperintensity	R Fr	L Fr/P/T/O	L T	R Fr/P	
DWI diffusion restriction	━	✝	✝	✝	
Cerebral sulcus and/or Meningeal enhancement	ND	ND	✝	ND	
Other abnormality	━	━	━	━	
EEG features					
Slow background activity	━	✝	━	✝	
Interictal spike wave	✝	━	✝	✝	
**Treatment latency (days)**	20	23	16	11	
Treatment					
Immunotherapy (IVIG, IVMP+OP)	✝	✝	✝	✝	
Anti-infective drugs	━	✝	✝	✝	
ASM	LEV	/	/	LEV LCM	
**Length of stay (days)** **Outcome**	23	25	35	19	
Follow-up duration (month)	38	22	26	4	
Recovery	✝	✝	✝	✝	
Seizure free	✝	✝	✝	✝	
Last cranial MRI/	Improved	Normal	Normal	Normal	
time interval(month)	/1	/6	/17	/1	
Last serum MOG-IgG/	1:10	1:10	━	━	
time interval(month)	/2	/8	/17	/1	
EDSS score (worse/last follow-up)	4.0/0	2.0/0	4.0/0	3.0/0	
Relapse	━	━	━	━	

ASM, anti-seizure medication; CSF, cerebrospinal fluid; DWI, Diffusion Weighted Imaging; EDSS, Expanded Disability Status Scale; F, female; Fr, frontal lobe; FBTCS, focal to bilateral tonic-clonic seizure; FLAIR, fluid-attenuated inversion recovery; FS, focal seizure; IVIG, intravenous immunoglobulin; IVMP, intravenous methylprednisolone; L, left; LCM, lacosamide; LEV, levetiracetam; LTG, lamotrigine; M, male; MOG, myelin oligodendrocyte glycoprotein; ND, no data available; O, occipital lobe; OP, oral prednisone; P, parietal lobe; PER, perampanel; R, right; T, temporal lobe; TS, tonic seizure; UTCS, unknown onset tonic-clonic seizure; WBC, white blood cell.

The predominant clinical symptoms were seizures (9/9, with 6 experiencing focal seizures), altered mental status (6/9), headache (5/9), fever (4/9), and focal neurological deficits (3/9, including hemiparesis and dysarthria). In addition, three children experienced status epilepticus, two had cranial nerve palsy (central facial paralysis and lingual paralysis), and two had Todd’s palsy. Notably, none of the children exhibited psychiatric/behavioral abnormalities, sleep disorders, visual impairments, or spinal cord lesions.

### Laboratory findings

The serum MOG-IgG titers ranged from 1:32 to 1:320. Of the nine patients, seven (7/9, 77.78%) exhibited CSF pleocytosis (median: 58/µL, range: 12-143/µL; normal ≤ 10/µL) with a predominance of lymphocytes, two patients had elevated CSF pressure (range: 240–280 mmH_2_O; normal value: 80–180 mmH_2_O), and one patient had slightly elevated CSF protein levels (0.46 g/L; normal value: 0.2-0.45 g/L). CSF-specific oligoclonal bands (OB) were detected in three patients(3/9). All patients had normal CSF glucose levels. Bacterial cultures and polymerase chain reaction (PCR) assays for herpes simplex virus (HSV), Epstein-Barr virus (EBV), cytomegalovirus (CMV), and mycoplasma pneumoniae (MP) in the CSF were negative in all patients.

### Electroencephalogram findings

An electroencephalogram (EEG) was conducted on all patients, revealing abnormal results in seven out of nine patients, including focal interictal discharges in six patients and slow background activities in three patients. During the monitoring period, three children experienced clinical seizures, including focal seizures with preserved awareness (patient 1 and patient 9) and focal-to-generalized tonic-clonic seizures (GTCS, patient 4). Additionally, one patient exhibited focal status epilepticus (patient 9).

### MRI findings

**Figure 1:**
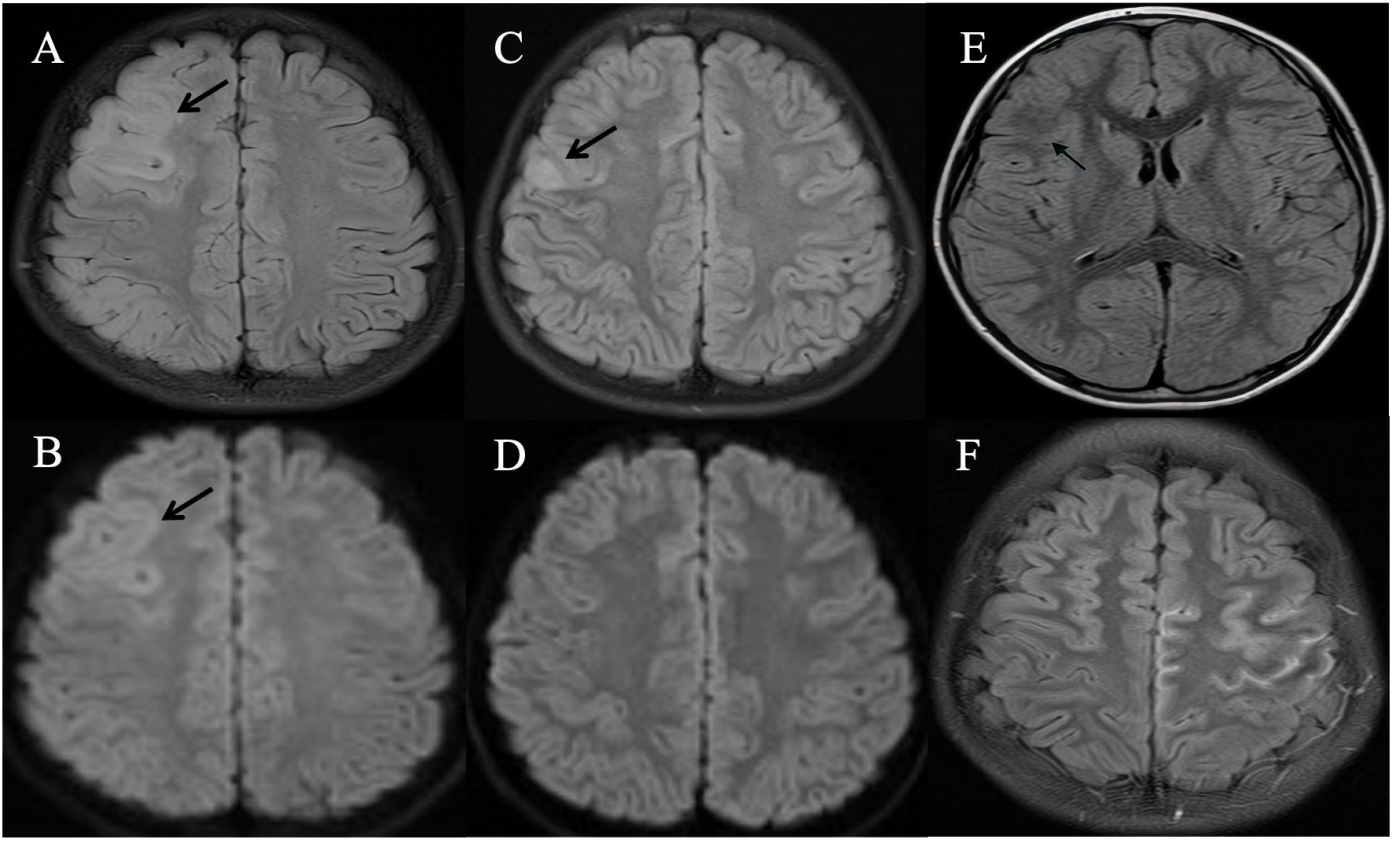
Brain magnetic resonance imaging (MRI) of patients with unilateral cortical FLAIR-hyperintense Lesion in Anti-MOG-associated Encephalitis with Seizures (FLAMES). **(A)** Fluid attenuation inversion recovery (FLAIR) image showed hyperintensity in the cortical region of the right frontal and parietal lobes, without white matter involvement (Patient 9). **(B)** Diffusion-weighted imaging (DWI) showed an obvious signal change in the cortex (Patient 9). **(C)** Hyperintensity in the cortical region of right frontal lobes in MRI FLAIR images (Patient 6). **(D)** DWI showed no obvious signal change in the cortex (Patient 6). **(E)** FLAIR image showed diminished signal intensity in the subcortical white matter at the lesion site in comparison to the contralateral hemisphere (Patient 3). **(F)** Gadolinium-enhanced T2-FLAIR imaging revealed multiple gyral enhancements in the left frontal and parietal cerebral cortex (Patient 5).

Brain MRI scans were performed on all nine patients, with the findings detailed in **Table 1**. FLAIR images revealed hyperintense lesions in all cases (**Figures 1A, C**), each showing unilateral cortical involvement (frontal lobe[8/9], parietal lobe[3/9], temporal lobe[2/9], and occipital lobe[1/9]). Seven children exhibited swelling in lesioned cortical regions. On diffusion-weighted imaging (DWI), only one child showed no diffusion restriction (**Figure 1D**), whereas the others showed varying degrees of diffusion restriction (**Figure 1B**). Additionally, in one case (**Figure 1E**), the signal in the subcortical white matter at the lesion site was lower than that on the contralateral side. Gadolinium-enhanced brain MRI examinations were conducted on five children, all of whom exhibited distinct enhancement of the corresponding cerebral sulci and/or meninges, precisely localized within the lesion cortex and its adjacent areas (**Figure 1F**).

It is notable that none of the nine patients showed involvement in the white matter or deep gray matter. Moreover, orbital and spinal cord MRIs were performed on all nine children, showing no abnormalities.

### Treatment and outcomes

The details regarding the treatment and outcomes of the nine patients are outlined in **Table 1**. The median time to initiate immunotherapy from symptom onset was 11 days (range, 4–23 days). Empirical anti-infective drugs were administered to seven children based on the suspicion of intracranial infection. Considering the risk of herpes simplex encephalitis and its potentially severe consequences, all seven children received acyclovir, with four also receiving antibiotics concurrently. Nevertheless, the anti-infective therapies proved ineffective for all patients.

Upon the definitive confirmation of MOGAD, immunotherapy was administered to all patients. In this study, the immunotherapy protocol consisted of intravenous immunoglobulin (IVIG, 1 g/kg/day for 2 days), intravenous methylprednisolone (IVMP, 20 mg/kg/day for 3 days with gradual tapering), and oral prednisone (OP, 1.5-2.0 mg/kg/day, tapered over 3–6 months). All nine children followed this immunotherapy regimen without any adjunctive immunosuppressive treatment.

Due to frequent seizures or epileptiform discharges, five children were prescribed single anti-seizure medications (ASM): two received perampanel, two received levetiracetam, and one received lamotrigine. Additionally, one child received combination therapy with lacosamide added to the existing regimen of levetiracetam. The median length of hospital stay was 23 days (range, 19–35 days). Notably, effective treatment led to a remarkable remission of symptoms in all patients at discharge.

### Follow-up

After discharge, all patients underwent outpatient follow-up. The median follow-up duration was 22 months (range, 4–38 months). Throughout this period, none of the children experienced relapse. The median time interval between the initial and last cranial MRI scans was 5 months (range, 1–33 months). Among these, four children exhibited a return to normal scans, and the abnormal signal ranges in five children significantly diminished. At the last follow-up, each child achieved favorable outcomes (EDSS score< 1.5, **Table 1**) and remained seizure-free. Among the six children who initially received ASM, two have had their medications discontinued.

## Discussion

There were overlapping clinical features; however, crucial radiological and pathological differences distinguished MOGAD from multiple sclerosis (MS) and aquaporin-4-IgG seropositive neuromyelitis optica spectrum disorder (AQP4-IgG NMOSD). Consequently, MOGAD has been confirmed as a distinct disease entity. Previous studies have indicated that symptoms resembling encephalitis, often accompanied by seizures, are more prevalent in MOGAD than in AQP4-IgG NMOSD (8). Among patients with MOGAD, 20.5% experience seizures (9), and 20.7% present with encephalitis, with 73% showing cortical involvement on brain MRI (10). Wang et al. reported that 16.9% (40/236) of pediatric patients with MOGAD exhibited less common phenotypes, with 6.8% (16/236) presenting as cortical encephalitis (11), a finding consistent with our study (7.32%). Notably, cortical encephalitis was identified as the predominant rare phenotype in pediatric MOGAD. While recent studies indicate that cortical encephalitis occurs more frequently in children than in adults (9% vs. 2%, 10% vs. 2.6%) (12, 13), there is a scarcity of case series studies dedicated to the pediatric population. Therefore, more attention is warranted to better understand this particular phenotype in children.

“FLAMES” represents a novel burning entity within MOGAD and is characterized by an atypical clinical phenotype. In 2019, based on the detailed description of 20 patients (including one pediatric case) and a comprehensive systematic literature review, Budhram named this rare phenotype, which exhibits distinct clinico-radiographic characteristics: in addition to epileptic seizures, over half of the patients presented with symptoms such as fever, headache, and those indicative of cortical lesions (including aphasia, motor impairment, and mental-behavioral disorders); brain MRI scans revealed cortical FLAIR hyperintensity, predominantly affecting one hemisphere. Interestingly, a proportion of patients (55%) had a prior history of behavioral changes or abnormal mental states (6, 14).

In this study, the median age of the children was 9 years, consistent with a recent study (15), suggesting an age-dependent nature of MOG-Ab-associated encephalitis. The seizure patterns were predominantly focal (6/9, 66.7%), which differed from the findings of other studies proposing that focal to bilateral tonic-clonic seizures are more common in FLAMES (16, 17). Among the nine children, four (4/9, 44.4%) experienced cluster seizures and three (3/9, 33.3%) exhibited focal status epilepticus. Based on the previous history, patient 9 was diagnosed with recurrent epilepsy and brain damage resulting from status epilepticus. In line with previous reports (18), for children with status epilepticus, especially those with a pre-existing diagnosis of epilepsy, cortical MRI alterations could be easily mistaken for prolonged seizure status (PSS) or epileptic brain injury. Therefore, it is crucial to consider the potential risk of FLAMES in children with epilepsy who experience recurrences without identifiable triggers, particularly those showing cortical lesions in cranial MRI. Four children (4/9, 44.4%) experienced fever, with one child experiencing prolonged and recurrent fever that was unresponsive to antibiotic therapy. Udani (19) reported that 12 children diagnosed with MOGAD presented with prolonged fever (PF), categorized as fever of unknown origin (FUO), aseptic meningitis (AM), and PF along with established acute demyelinating syndrome (ADS). In children, PF accompanied by significant inflammatory markers requires consideration of MOGAD. Consistent with the findings of Budhram, each child exhibited no less than two symptoms. It is noteworthy that the non-specificity of symptoms might give rise to diagnostic uncertainty. Moreover, clinical and/or radiological demyelinating events may occur prior to, concurrently with, or following cortical encephalitis (10, 20). In general, MOGAD typically presents with a gradual and monophasic clinical course, especially in children.

The MRI findings in MOG encephalomyelitis typically present an acute disseminated encephalomyelitis (ADEM)-like pattern, characterized by diffuse signal changes in the cortical grey matter, as well as in the juxtacortical/subcortical/deep white matter, and deep grey matter, as evidenced by FLAIR images. Furthermore, the MRI results may either be normal or reveal non-specific gray matter lesions (including the cortex, basal ganglia, thalamus, brainstem, and cerebellum), with the white matter being spared. Notably, the most prevalent MRI pattern associated with seizures in MOGAD is Cerebral Cortical Encephalitis (CCE), with unilateral involvement occurring more frequently than bilateral (21). Cortical hyperintensity can affect any lobe of the brain, with the frontal lobes being the most commonly involved (11). A prior cohort study on MOGAD-CCE revealed that 53% of patients exhibited concomitant cerebral parenchymal T2 hyperintensities on MRI (22). Notably, when establishing a phenotypic diagnosis, it is necessary to carefully evaluate the presence of multifocal clinical phenotypes, including cerebral monofocal or polyfocal deficits, ADEM, or the leukodystrophy-like phenotype. The primary aim of this study is to investigate the clinical characteristics of “pure cortical involvement” in CCE. Excluding findings associated with multifocal lesions or phenotypes may enhance the reliability of the study’s conclusions. In this study, all nine children exhibited unilateral cortical involvement, with the frontal lobes being predominantly affected (8/9), which was consistent with previously mentioned findings. Some studies (6, 23) have indicated that approximately 20% of FLAMES patients have bilateral cortical involvement, which may be associated with more widespread and severe clinical manifestations, such as critical illness, severe encephalopathy, refractory epilepsy, and worse outcomes. Moreover, five children (5/9, 55.6%) showed corresponding cerebral sulcus and/or meningeal enhancement, a finding regarded as a characteristic feature in children (24), which has prompted some scholars to postulate the existence of a primary meningeal disorder. Therefore, it is crucial to recognize that T2-FLAIR hyperintensity, although characteristic of FLAMES, is not diagnostic on its own and necessitates careful differentiation from conditions such as meningoencephalitis, metabolic encephalopathy (including hypoglycemia, hyperammonemia, or hypoxemia), and mitochondrial encephalomyopathy. Importantly, certain supplementary imaging markers might potentially be valuable for identifying FLAMES, including the decreased signal in the subcortical white matter at the lesion site compared to the contralateral side (24), distinct characteristics of the corresponding cortical lesion area such as intravascular and perivascular multiple grainy enhanced lesions (25), hyperperfusion on Single-Photon Emission Computed Tomography (SPECT), and decreased oxygen extraction on Susceptibility Weighted Angiography (SWAN) (17).

Cerebrospinal fluid pleocytosis was observed in 95.2% of patients with FLAMES, with a minority displaying elevated CSF pressure and protein levels. In this study, seven children (7/9, 77.8%) had CSF pleocytosis with lymphocytic predominance, one had elevated CSF protein, and two had elevated CSF pressure, which could be life-threatening. Currently, it remains unclear whether the titer of MOG antibodies correlates with the severity of disease and the risk of recurrence. Although there appears to be a positive association between higher antibody titers and the duration of headache and fever, seizure frequency, and visual impairment, statistical evidence is lacking. Some data suggest that persistent MOG-antibody positivity and bilateral cortical FLAIR hyperintensity involving the medial frontal lobes might be potential risk factors for recurrence (26).

In this study, seven pediatric patients received anti-infective treatment initially due to suspected viral encephalitis. However, no significant clinical symptom improvement was observed until immunotherapy was initiated. The low incidence, non-specific clinical manifestations, and insufficient related literature have contributed to the current absence of evidence-based treatment guidelines for FLAMES. Recent research has indicated that immunotherapy demonstrates high efficacy for patients with this condition. The recommended first-line treatment involves corticosteroids, typically initiating with a high-dose intravenous infusion of methylprednisolone, followed by an oral prednisone taper. Extending the duration of treatment and gradually decreasing the dosage of oral corticosteroid dosage could significantly reduce the rate of relapse, with a nearly 50% decrease observed (from 47% to 25%). For severe cases or those with a poor response to corticosteroids, second-line therapies such as intravenous immunoglobulins and plasmapheresis may be considered. For patients unresponsive to the aforementioned therapies or experiencing recurrent relapses, immunosuppressive therapy could be considered as the subsequent treatment option. In our case series, all children demonstrated a positive response to immunotherapy and achieved a satisfactory outcome, without necessitating immunosuppressive intervention. However, brain MRI scans of five children remained abnormal at the last follow-up, indicating that MRI cortical abnormalities might persist for months or even longer after the resolution of clinical symptoms.

In comparison to antiseizure medications (ASMs), immunotherapy has been proven to be more effective in controlling acute seizures. There have been reports indicating that, in certain cases, seizures could be completely resolved with steroids alone. However, in children with recurrent seizures or status epilepticus, it might be challenging to accurately assess the clinical value of early ASMs administration. In this study, all participants had achieved seizure-free status by their last follow-up. Five of the nine cases were administered with ASM monotherapy, and one pre-diagnosed epilepsy case required a supplementary ASM in combination with the original monotherapy. It is noteworthy that cases of refractory epilepsy and even super-refractory status epilepticus have been documented (21, 27). The long-term risk of epilepsy in children with cerebral cortical encephalitis might be more variable than previously presumed. Additionally, the optimal duration of ASM treatment remains undetermined.

MOG, a glycoprotein located on the outermost layer of oligodendrocytes, constitutes an essential component of myelin. Current autopsy and biopsy results from patients with MOGAD demonstrate a CD4+ T cell response, accompanied by granulocyte inflammation. These findings indicate that MOGAD is an antibody-mediated central nervous system (CNS) demyelinating disease. In contrast, FLAMES represents an autoimmune encephalitis without demyelinating lesions, with its underlying pathological mechanism still unresolved. In recent years, an increasing number of reports have described cortical encephalitis and meningoencephalitis with positive MOG antibodies, despite the absence of demyelinating lesions. As a result, some scholars have proposed that anti-MOG antibodies may not have a direct correlation with non-demyelinating encephalitis, and there could be other autoimmune encephalitis-related antibodies coexisting with MOG antibodies. Furthermore, pathological findings have revealed inflammatory changes in both the cortex and subcortex, with microglial proliferation in the perivascular regions and subcortical white matter, a discovery that potentially indicates the presence of “preactive” demyelinating lesion (28).

This study had some limitations. Firstly, it was a single-center, retrospective study with a limited sample size, which highlights the necessity of conducting a prospective study with a larger cohort, particularly to explore the influencing factors of clinical outcomes and relapse. Secondly, the follow-up duration was relatively short. A more prolonged follow-up is essential to ascertain the actual recurrence rate of FLAMES.

## Conclusion

FLAMES has emerged as a distinct sub-entity within the MOGAD spectrum and is recognized as the predominant rare phenotype in pediatric patients. In the pediatric population, the presence of specific features warrants consideration of FLAMES, necessitating prompt MOG antibody testing. These features include school-age children, focal epileptic seizures, FLAIR-hyperintense cortical lesions observed on cranial MRI, especially when coupled with concurrent enhancement of the corresponding cerebral sulci and/or meninges.

In addition, for children suspected of encephalitis showing no response to anti-infective therapy, as well as those experiencing epilepsy recurrence without identifiable triggers, especially when cranial MRI reveals cortical lesions, vigilance for MOG-Ab associated encephalitis is warranted. Future research with larger cohorts is essential to identify specific clinical characteristics, factors affecting disease progression, optimal therapeutic approaches, and long-term outcomes.

The study was approved by the Medical Ethics Committee of Hebei Children’s Hospital, affiliated with Hebei Medical University. Informed consent was obtained from the parents or legal guardians of the participating children.

## Data Availability

The raw data supporting the conclusions of this article will be made available by the authors, without undue reservation.
